# Immune Regulation of Seminal Plasma on the Endometrial Microenvironment: Physiological and Pathological Conditions

**DOI:** 10.3390/ijms241914639

**Published:** 2023-09-27

**Authors:** Qiuzi Shen, Xiaoyu Wu, Jin Chen, Chao He, Zehao Wang, Boyan Zhou, Huiping Zhang

**Affiliations:** 1 Institute of Reproductive Health, Tongji Medical College, Huazhong University of Science and Technology, Wuhan 430030, China; sqz@hust.edu.cn (Q.S.); 15735179602@163.com (X.W.); chenjinCJD@163.com (J.C.); m202175762@hust.edu.cn (C.H.); 2 School of Management, Huazhong University of Science and Technology, Wuhan 430074, China; m202177730@hust.edu.cn

**Keywords:** seminal plasma, endometrium, immune cell, cytokines, immune tolerance, embryo implantation, pregnancy

## Abstract

Seminal plasma (SP) accounts for more than 90% of semen volume. It induces inflammation, regulates immune tolerance, and facilitates embryonic development and implantation in the female reproductive tract. In the physiological state, SP promotes endometrial decidualization and causes changes in immune cells such as macrophages, natural killer cells, regulatory T cells, and dendritic cells. This leads to the secretion of cytokines and chemokines and also results in the alteration of miRNA profiles and the expression of genes related to endometrial tolerance and angiogenesis. Together, these changes modulate the endometrial immune microenvironment and contribute to implantation and pregnancy. However, in pathological situations, abnormal alterations in SP due to advanced age or poor diet in men can interfere with a woman’s immune adaptation to pregnancy, negatively affecting embryo implantation and even the health of the offspring. Uterine pathologies such as endometriosis and endometritis can cause the endometrium to respond negatively to SP, which can further contribute to pathological progress and interfere with conception. The research on the mechanism of SP in the endometrium is conducive to the development of new targets for intervention to improve reproductive outcomes and may also provide new ideas for semen-assisted treatment of clinical infertility.

## 1. Introduction

Semen is a complex fluid, composed of spermatozoa and seminal plasma (SP). SP consists of secretions from the male accessory gonads, including the seminal vesicle gland, prostate, and bulbourethral gland, as well as testis and epididymis [1]. SP provides sperm to fertilize the oocyte at conception and also contributes to reproductive success through complex interactions with the woman’s reproductive and immune systems after fertilization [2,3]. SP contains a range of biologically active signaling factors, including cytokines, prostaglandins (PGs), sex steroid hormones, glycans, nucleic acids, and other small molecules in soluble form that are encapsulated in extracellular vesicles or associated with spermatozoa [4,5,6]. Once in the female reproductive tract, cytokines and other signaling molecules of SP interact with sperm in semen [7] to act together to modify the transcriptional program of the female reproductive tract, causing molecular and cellular changes that promote embryo development and implantation [2,8]. It can increase the chances of bearing offspring and pass on male genes to the next generation [9]. This interaction exists in almost all species that deposit male gametes via semen injection into the female reproductive tract [3] and will have a long-term impact on the survival and health of future generations [8,9,10,11].

The female responses triggered by SP are composed of alterations in gene transcription and cellular function in the cervix, uterus, fallopian tube, ovary, and uterine-draining lymph node. They are reflected in the following aspects: (1) in the cervix and uterus, removing microorganisms and excess sperm introduced during copulation; (2) in the ovary, promoting ovulation, corpus luteum formation, and progesterone synthesis; (3) in the fallopian tube, inducing cytokines that regulate the development of the preimplantation embryo and support the storage of sperm; (4) in the uterus, facilitating leukocyte recruitment, thereby inducing endometrial capacitation and facilitating embryo implantation; (5) in the uterine-draining lymph node, initiating an adaptive immune response, which promotes the production of regulatory T cells (Tregs) and mediates the tolerance to paternal graft antigens. These responses interact with endometrial events and together promote oocyte fertilization and embryo implantation [9].

This paper mainly reviewed the compositions of SP and their immune regulation on the endometrial microenvironment, especially the pathological effects of SP on endometrium and pregnancy. We look for new male factors that affect pregnancy outcomes to provide new ideas and intervention targets for the semen-assisted treatment of clinical infertility.

## 2. Methods

A systematic search was conducted in PubMed and Web of Science databases from inception to July 2023. For the part of SP components, we used the following query: ((“seminal plasma”[Title/Abstract]) OR (“semen”[Title/Abstract]) OR (“sperm”[Title/Abstract])) AND ((“cytokine”[Title/Abstract]) OR (“exosome”[Title/Abstract]) OR (“extracellular vesicle”[Title/Abstract]) OR (“proteome”[Title/Abstract]) OR (“transcriptome”[Title/Abstract]) OR (“metabolome”[Title/Abstract])). A total of 1792 records were retrieved.

For the part of the impact of SP on the immune microenvironments of the endometrium, we used the following query: ((“seminal plasma”[Title/Abstract]) OR (“semen”[Title/Abstract]) OR (“sperm”[Title/Abstract])) AND ((“endometrium”[Title/Abstract]) OR (“endometrial”[Title/Abstract]) OR (“female genital tract”[Title/Abstract])) AND ((“immune”[Title/Abstract]) OR (“cytokine”[Title/Abstract]) OR (“immune cell”[Title/Abstract])). A total of 211 results were retrieved. Other articles were extracted from the reference lists of the articles found by entering the aforementioned keywords.

Both animal and human studies were considered suitable for this review. After screening the title and abstracts, non-mammalian studies and articles without clearly describing the species were excluded.

## 3. Components of Seminal Plasma (SP)

**Figure 1:**
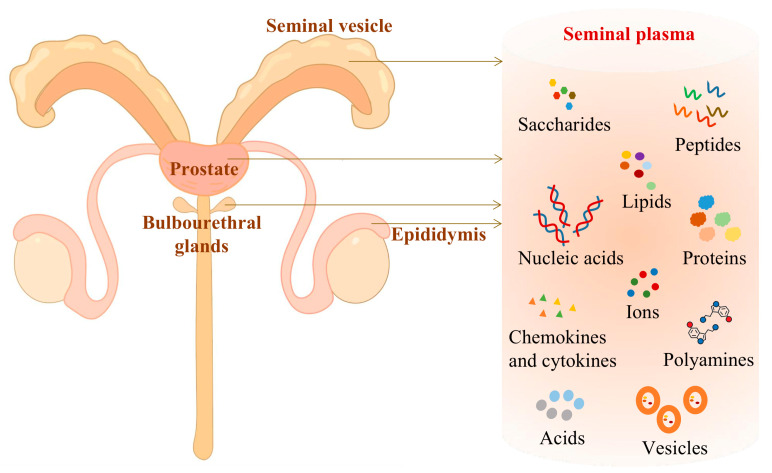
Source and main components of seminal plasma (SP). The SP mainly comes from the seminal vesicle, prostate, epididymis, and bulbourethral gland. It includes water, saccharides, lipids, proteins, ions, nucleic acids, polyamines, peptides, chemokines, cytokines, vesicles, organic acids, inorganic acids, etc.

SP, the noncellular component of semen, comprises 95% of the total volume of semen. As shown in Figure 1, about 90% of SP is produced by the accessory gonads, with a small percentage coming from the bulbourethral gland and epididymis [12]. The composition of SP is complex, including water, saccharides (fructose, glucose, galactose, and mannose), lipids (cholesterol and testosterone), a large number of complex proteins of unknown function, ions (zinc, calcium, and citrate), nucleic acids, polyamines, and peptides [1,12]. In addition to these components, SP contains chemokines, cytokines, and PGs [13], including the proinflammatory factors interleukin (IL)-1β, IL-8, tumor necrosis factor (TNF)-α, interferon (IFN)-γ, leukemia inhibitory factor (LIF), IL-6 [14]; immunomodulatory factors IL-10, transforming growth factor (TGF)-β [15]; vascular endothelial growth factor (VEGF), epidermal growth factor (EGF), and granulocyte-macrophage colony stimulating factor (GM-CSF), which affect vascular growth [16]. These factors play an important role in inducing inflammation, modulating immune tolerance, and promoting early embryonic growth and implantation. In addition, SP contains a large number of extracellular vesicles (EVs) released by accessory glands, containing proteins, DNA, RNA, and lipids [17], which have the ability to regulate endometrial inflammation [18] and the uterine environment to promote sperm survival and activation [19].

### 3.1. The Proteome in SP

Although there are differences in protein type and origin between species, it is possible to categorize the major proteins in SP into three types: protein carrying the fibronectin-2 (Fn-2), sperm adhesin, and cysteine-rich secretory protein (CRISP) [20]. Sperm adhesion proteins are multifunctional 12–16 kDa glycoproteins and can be categorized as heparin-binding proteins (HBPs) and non-binding proteins based on whether they bind heparin or not. Sperm adhesion proteins influence several aspects of spermatozoa, including membrane stabilization, capacitation, and interactions between sperm–oviduct intima or sperm–zona pellucida [21,22,23]. SP also contains a large number of proteases, including phosphatases, aminopeptidases, glycosidases, and matrix metalloproteinases [24,25,26], which play an important role in male fertility [27,28]. In addition, SP contains protein compounds similar to plasma proteins, such as prealbumin, albumin, α-, β-, and γ-globulins, transferrin, immunoglobulins, complement factors, and cytokines [29,30,31]. Differences in cytokine expression between species and individuals may be related to inflammation in the male reproductive tract [32] and the number of exfoliated leukocytes [33,34].

### 3.2. The Metabolome in SP

Metabolomics is an emerging discipline for the qualitative or quantitative analysis of metabolites in specific components of organisms, which focuses on small molecules with molecular weights less than 1000, such as amino acids, peptides, fatty acids, sugars, and inorganic salts. Metabolite screening has demonstrated its application in identifying potential markers of male fertility and infertility. Kavanagh used nuclear magnetic resonance hydrogen spectroscopy (^1^H-NMR) to determine metabolites in human SP and indicated that citrate is one of the most abundant metabolites in human SP [35] and is also a marker for a variety of semen pathologies (azoospermia, oligospermia, teratospermia, asthenospermia, and oligoasthenoter atozoospermia) [36,37,38]. Menezes et al. utilized the gas chromatography–mass spectrometer (GC-MS) to study 22 metabolites in Holstein bull SP with different fertility and showed that organic acids and fatty acids were the most prevalent metabolite classes in bull SP. The most abundant metabolites detected were phosphoric acid, oleic acid, carbamates, glycerol, and phosphorus, and the least abundant were acetic acid, L-serine, 2-ketobutyrate benzoic acid, and carbonate [39]. In another study on Holstein bulls, metabolites such as 2-oxoglutarate and fructose differed significantly between high and low-fertility groups, revealing the value of 2-oxoglutarate and fructose as potential markers of fertility in bulls [40].

In addition, several studies have revealed possible functions of SP metabolites. Cholesterol was detected in spermatozoa, which is important in regulating sperm membrane permeability and fluidity. Carnitine has an important role in fatty acid oxidation and β-oxidation in spermatozoa [41]. Zhang et al. analyzed the metabolomics of boars SP by ultra-high-performance liquid chromatography coupled with quadrupole time-of-flight mass spectrometry (UHPLC-qTOF-MS). A total of 953 metabolites were analyzed, of which 50 showed significant variations between the high and low freezing resistance groups. In addition, 12 metabolites were analyzed for metabolic targets, and D-aspartate, N-acetyl-L-glutamic acid (NAG), and inosine showed differences. Thus, D-aspartate, NAG, and inosine in SP may be potential markers for assessing boar sperm cryopreservation resistance [42].

## 4. Immune Regulation of Endometrial Microenvironment by SP under Physiological Conditions

The endometrium undergoes cyclic shedding, regeneration, and differentiation throughout the menstrual cycle as it is hormonally regulated by the hypothalamic–pituitary–ovarian axis. The proportion and function of immune cells in the endometrium during this process also change in response to hormone levels [43]. CD68^+^ macrophages are present at any time during the menstrual cycle, and the number of macrophages increases significantly during the secretory phase, especially at the site of implantation [44,45]. The density of CD1a^+^ immature dendritic cells (DCs) is significantly higher than that of CD83^+^ mature DCs throughout the menstrual cycle [44]. Natural killer (NK) cells account for a small percentage during the proliferative phase but could surge to 70% of endometrial leukocytes by the late secretory phase [46].

### 4.1. Immune Responses to Endometrial Exposure to SP

#### 4.1.1. Decidualization

In preparation for implantation, the endometrium undergoes decidualization, a process that allows the endometrium to differentiate into morphologically and functionally distinct pregnancy tissues called decidua. The decidua is characterized by a dense cellular matrix of polygonal cells, which are produced by the differentiation of fibroblast-like endometrial stromal cells (ESCs) [47]. The success of decidualization depends on two main criteria. First, the decidualization must be precisely timed to the window of implantation. Second, ESCs must be sufficiently decidualized to accommodate the implanted blastocyst. Uterine decidualization occurs under the influence of the elevated postovulatory sex steroid hormone progesterone (P_4_) [47]. In the absence of decidualization, pregnancy cannot be maintained [48,49]. Thus, an inadequately decidualized endometrium and the consequent decrease in receptivity are major fertility-limiting factors [50,51].

Doyle et al. demonstrated that SP can act as a specific decidualizing agent in the human endometrium. SP enhanced and accelerated the P_4_-mediated decidualization of human ESCs and may enhance endometrial receptivity [47]. ESCs treated with a combination of SP and P_4_ underwent phenotypic differentiation, exhibited polygonal morphologic features of metamorphosis, and induced metamorphosis-specific markers prolactin (PRL) and insulin-like growth factor binding protein 1 (IGFBP1) at both the mRNA and secreted protein levels. The active components of SP can be used in combination with P_4_ as a clinical decidualizing agent to complement natural and assisted reproductive therapies [47].

George et al. demonstrated that the ability of SP to promote decidualizing depended on IL-11 and that SP amyloid was not a potent signaling agent [52]. A fraction of SP enriched in seminal microvesicles (MVs) promoted decidualization in human primary endometrial stromal fibroblasts (eSFs), but whether MVs fused with eSFs to transmit decidualizing signals was not known. SP proteins that promote decidualization induced the transcriptional responses related to cell differentiation in eSFs. However, the SP proteins responsible for initiating and enhancing eSF decidualization and the cellular mechanisms of SP-induced decidualization still need to be further investigated [52]. Interestingly, SP also promoted the decidualization of eSFs in women with polycystic ovary syndrome (PCOS) and endometriosis, but the exact mechanism was unknown. It seems to be inconsistent with the notion that SP contains proinflammatory cytokines and normally triggers an inflammatory response in cells of the female reproductive tract. George et al. hypothesized that SP would not amplify the inflammation already present in eSFs of women with PCOS or endometriosis, but rather trigger a different type of response that promotes decidualization [52].

Rodriguez-Caro et al. found that seminal fluid extracellular vesicles (SF-EVs) were able to bind to human ESCs in vitro, enhancing ESC decidualization and increasing PRL secretion, which may improve endometrial receptivity [53]. In the future, it is possible to evaluate whether SF-EVs can be used for in vitro fertilization (IVF) therapy [53].

#### 4.1.2. Changes in Immune Cells

Mouse SP signaling delivers paternal antigens that stimulate the proliferation and recruitment of Tregs [54,55,56]. Tregs promote endometrial tolerance production by inhibiting and alleviating inflammation and supporting fetal and optimal placental development [57,58]. NK cells, the most abundant leukocyte population in the endometrium during the implantation window, can express a range of growth factors involved in angiogenesis in embryo implantation, including VEGF-C, placental growth factor, and angiopoietin-2 [59,60]. After SP exposure, the number of CD56^+^ NK cells increased in the endometrium and CD57^+^ NK cells increased in the ectocervix [15,61]. Macrophages in the decidualization are classified as M2 phenotype and exhibit immunosuppressive properties characterized by a high production of IL-10 and indoleamine 2,3-dioxygenase activity. These characteristics favor maternal immune tolerance to the embryo [62]. Under the influence of PGs and other inflammatory mediators of SP, macrophages produce large amounts of matrix metalloproteinases, which are secreted prior to implantation and play an important role in embryo implantation and placental development [63]. DCs are the predominant antigen-presenting cells and are able to modulate adaptive immune responses by presenting antigens and instructing T cells to acquire immunomodulatory or effector phenotypes. During embryo implantation, DCs are recruited to the implantation site in response to SP exposure. The depletion of uterine DCs during implantation leads to the disruption of vessel formation and subsequent impaired implantation. In addition, uterine DCs mediate tolerance to paternal antigens expressed by the developing embryo by taking up SP alloantigens and presenting them to T cells [64,65,66].

#### 4.1.3. Formation of Neutrophil Extracellular Traps (NETs)

In the bovine reproductive system, semen is naturally deposited into the vagina. As the sperm migrates through the cervix into the uterus, most of the SP is left behind [67]. However, during artificial insemination (AI), sperm, SP, and components such as diluents and cryoprotectants are transferred to the uterus, which may alter the early innate immune response [68]. The first cellular response to nonadaptive immunity in mammals is the rapid influx of polymorphonuclear neutrophils (PMNs) into the uterine cavity. PMNs migrate to the site of infection and inactivate and kill pathogens by phagocytosis, the secretion of antimicrobial substances, and the release of neutrophil extracellular traps (NETs) [68]. The formation of NETs (ETosis [69]) is not only a novel mechanism of PMNs, but also a novel mechanism of monocytes, eosinophils, mast cells, and macrophages to fight bacteria, fungi, viruses, parasites, and spermatozoa [67,70,71,72,73,74,75,76,77,78,79,80]. NETs are formed through complex mechanisms involving multiple receptors and signaling pathways [72,80,81,82,83].

Studies have shown that SP influences NET formation [67,70,84,85]. Alghamdi et al. found that sperm-induced NETosis was different between cattle and horses, particularly depending on the presence or absence of SP [67]. Both equine sperm and SP were deposited directly in the uterus. Certain components of SP, such as deoxyribonuclease (DNase), appeared to inhibit sperm binding to neutrophils, thus preventing NET formation and increasing fertility [67,70,85], whereas bovine sperm showed a low binding to PMNs in the absence of SP, and the addition of 10% SP increased sperm-neutrophil binding and NET formation [67]. Bovine DNase from SP and SP proteins did not digest the sperm–neutrophil network [86]. Fichtner et al. also demonstrated that bovine SP alone induced NET formation in vitro in a concentration-dependent manner in the absence of any sperm. The ability of SP to induce NETs was lower in older bulls compared to younger bulls [68]. However, so far, the relationship between NET formation and fertility has not been clarified, so further in vivo evidence is needed [68]. Since NET formation is associated with reduced sperm motility [80,87,88], the researchers speculate that it may lead to decreased fertility [68].

#### 4.1.4. Secretion of Cytokines

SP has been thought to be a transport mediator for sperm crossing the female reproductive tract [4]. However, in animal studies, it has been gradually discovered that SP also transmits a series of signaling molecules to females and interacts with the female reproductive tract epithelial cells and immune cells to trigger a local inflammatory response in the reproductive tract [2]. In mice, signaling factors in SP bind to receptors expressed by epithelial cells in the lining of the uterine cavity and activate the synthesis of GM-CSF [89,90]. This is followed by an immediate elevation of several cytokines and chemokines, including *Il-6*, *Tnf*, C-X-C motif chemokine ligand 1 (*Cxcl-1*), C-X-C motif chemokine ligand 2 (*Cxcl-2*), C-C motif chemokine ligand 3 (*Ccl3*), and granulocyte colony stimulating factor (*Gcsf*) [91,92,93,94,95]. It has been demonstrated that the signaling molecule in mouse SP is predominantly TGF-β [96,97]. However, although high levels of TGF-β were similarly detected in porcine SP [98] and the immunosuppressive activity of TGF-β was correlated with appropriately sized protein fractions in boars [99], it has not been demonstrated whether this family of cytokines contributes to the modulation of inflammatory responses in the reproductive tract of sows.

Exposure of bovine endometrial epithelial cells to SP resulted in the increased expression of *GM-CSF*, *IL-8*, transforming growth factor beta 1 (*TGFB1*), prostaglandin-endoperoxide synthase 2 (*PTGS2*, also known as cyclooxygenase-2, *COX-2*), and aldo-keto reductase family 1 member C4 (*AKR1C4*). Exposure of ESC to SP resulted in an increased *GM-CSF*, *IL1B*, *IL6*, *IL-8*, *IL17A*, *TGFB1*, *PTGS2*, and *AKR1C4* expression [100]. In pigs, SP induced the expression of *GM-CSF*, *IL-6*, monocyte chemoattractant protein 1 (*MCP-1*), and *COX-2* [101]. Studies in horses and sheep similarly showed that SP exposure induced endometrial cytokine expression, with an increased expression of *IL-1B*, *IL-6*, *TNF-α*, and *COX-2* in horses [102] and an increased expression of GM-CSF and IL-8 in sheep [103]. SP also induced IL-17A secretion by γδT cells. The blockade of IL-17A decreased the number of uterine neutrophils and prevented their migration to epithelial cells by decreasing the expression of the chemokines *Cxcl1*, *Cxcl2*, and C-X-C motif chemokine ligand 5 (*Cxcl5*). Blockade of IL-17A did not affect Th1/Th2 balance but actually attenuated inflammation in the uterus by reducing the expression of *Il-1β* and *Tnf-α* [104]. Thus, IL-17A may be an important intermediate cytokine in SP leading to endometrial inflammation.

#### 4.1.5. Changes in the Expression Profile of Secreted miRNA

There is abundant evidence that reproduction is accompanied by alterations in the expression of a large number of miRNAs. The male and female miRNA-deficient mice bred by the inhibition of Drosha and Dicer enzymes exhibited sterility [105,106]. A study found that semen led to an increase in several miRNAs in the reproductive tract of female mice. The increased miRNAs included miR-223 and miR-146a, which are associated with immune tolerance. And miR-155 is expressed in uterine tissues and draining lymph nodes and may contribute to the regulation of DC function and Treg behavior [107]. Another study in mice showed that SP interacted with endometrial epithelial cells and resulted in the differential expression of 225 genes, including 190 upregulated and 145 downregulated, with many differentially expressed non-coding miRNAs. Many of the differentially expressed mRNAs and miRNAs have known roles in the immune response, leading to a range of cytokines and chemokines that are synthesized locally [90].

#### 4.1.6. Changes in Gene and Protein Expression

The interaction between SP and the endometrium regulates the expression of genes related to endometrial receptivity in mice [108] and affects endometrial proliferation, differentiation, decidualization, and angiogenesis [109,110,111]. In the porcine model, SP inhibited the mRNA expression of endometrial *PTGS2* and stimulated the expression of maturation-enhancing factors, including mitogen-activated protein kinase 1 (*MAPK1*) [112]. However, in the bovine model, SP did not come into direct contact with the endometrium of the cow due to species specificity. Therefore, it has been shown that it is the sperm but not the SP that leads to a differential expression of endometrial genes in cattle [113].

PGs are critical for embryo implantation and the ability to achieve a successful pregnancy [48,114,115]. SP modulates the expression levels of prostaglandin E_2_ (PGE_2_) and prostaglandin F_2α_ (PGF_2α_) in the uterine lumen and endometrium [116,117], thereby altering the uterine environment and affecting embryo development and attachment in early pregnancy.

### 4.2. Regulation of Endometrial Microenvironment by SP Signaling Factors

#### 4.2.1. Transforming Growth Factor (TGF)-β

TGF-β family cytokines synthesized in seminal vesicle glands have been identified as key signaling factors of SP [96,118]. In mice, TGF-β and unidentified Toll-like receptor-4 (TLR4) ligands of SP bound to receptors on endometrial epithelial cells, triggering a transcriptional program that led to a surge in proinflammatory cytokine and chemokine synthesis, as well as an influx of leukocytes into endometrial tissue [89,90,96,119]. In human samples, TGF-β3 caused changes in the expression of several proinflammatory cytokines and chemokines in human Ect1 cervical epithelial cells. All TGF-β isoforms (TGF-β1, TGF-β2, and TGF-β3) showed the ability to induce the mRNA and protein expression of GM-CSF and IL-1 in Ect1 cells, and several other cytokines present in SP did not elicit Ect1 cell responses. This suggests that all three TGF-β isoforms are key signaling factors in the SP for inducing proinflammatory cytokine synthesis in the human endometrium [118].

#### 4.2.2. Prostaglandins (PGs)

PGs in seminal plasma are secreted by the epithelial cells of the seminal vesicle glands and prostate. PGE_2_ and 19-OH-PGE are the major PGs in human SP [120,121]. Due to its immunosuppressive feature, 19-OH-PGE is hypothesized to protect male spermatozoa from immune damage and protect females from sperm antigens [122,123]. This was also confirmed in vitro using 19-OH-PGE to stimulate human cervical explants [124]. PGE_2_ promotes tumorigenesis and angiogenesis in endometrial epithelial cells through the activation of fibroblast growth factor 2 (FGF-2), COX-2, and VEGF expression, as well as epidermal growth factor receptor (EGFR) and extracellular-signal-regulated kinase (ERK) 1/2 signaling pathways [125]. The combination of PGs and E-prostaglandin receptor-2 and 4 promotes the differentiation of tolerogenic DCs, downregulates IL-12p70, IL-1β, TNF-α, and IL-6, and upregulates IL-10 and TGF-β expression to promote the endometrial inflammatory response, thereby favoring fertility [126].

#### 4.2.3. Interleukin (IL)-8

Considerable concentrations of IL-8 are present in SP [127]. A concentration-dependent increase in *IL-1β*, *IL-6*, and *LIF* mRNA expression was found in human endometrial epithelial cells incubated with 0.1%, 1%, and 10% SP in vitro. Using human recombinant IL-8 with the same concentration as that in SP in the physiological state could also stimulate the expression of *IL-1β*, *IL-6*, and *LIF* in human endometrial epithelial cells in vitro. This shows that SP stimulated the expression of proinflammatory cytokines in endometrial epithelial cells in vitro, and this effect may be at least partly exerted by a large number of IL-8 existing in SP, while the stimulatory effect of IL-8 alone on endometrial epithelial cells was slightly reduced compared with that of SP [128]. This indicates that cytokine expression in human endometrial epithelial cells in vitro is not regulated by a single cytokine alone but is co-regulated by multiple cytokines in SP (Table 1).

#### 4.2.4. Antigen

Semen contains several male-individual-specific antigens, including major histocompatibility complex (MHC) class Ia, Ib, and class II [129]. It can be presented by macrophages and DCs recruited to the endometrium [89,130], followed by antigen-presenting cells (APCs) transporting paternal antigens to the uterine, draining lymph nodes or interact with uterine T cells to drive the activation and expansion of the clonal subpopulation of Tregs. Tregs recognize paternal antigens and respond to them appropriately [131].

#### 4.2.5. Exosome

Typical semen ejaculated by mammals contains trillions of EVs, which are a major component of the SP [132]. EVs are membrane-enclosed complexes that facilitate intercellular communication through their contents, including proteins, lipids, and nucleic acids (RNA and DNA). The major types of EVs are (1) exosomes, 30–100 nm vesicles that are formed in the multivesicular bodies (MVBs) and released into the intercellular space by MVB fusion with the plasma membrane, (2) microvesicles, 100 nm–1 µm vesicles that are shed from the plasma membrane, (3) apoptotic bodies, vesicles of approximately 1–5 µm, and (4) large oncosomes, vesicles secreted by cancer cells [133]. In the male reproductive tract, EVs are produced by the male accessory glands, including the seminal vesicle, prostate [134], and epididymis [135], and are present in the semen [134]. EVs in semen have a known role in enhancing sperm function [53]. SP exosomes (sExos) and SP provide immunomodulatory functions in the uterus [18], enhance the process of ESC decidualization, and modulate the release of PRL [53]. sExos modulation of the immune response and gene expression in the female reproductive tract ultimately contributes to embryo implantation and pregnancy [19,52,136], and later regulates embryonic development [137].

#### 4.2.6. Other Signal Factors

VEGF is a heparin-binding homodimeric glycoprotein, a mitogen for endothelial cells, and a potent inducer of angiogenesis [138] that also promotes vascular permeability [139]. It is found in abundance in SP [128] and plays a role in implantation regulation and endometrial angiogenesis [140]. Other novel signaling molecules are sperm adhesin porcine seminal protein (PSP)-I/PSP-II, which contribute to neutrophil and T cell recruitment in the porcine uterus [141] and maintain sperm viability, motility, and mitochondrial activity in vitro [142]. The role of CRISP-3 in regulating the endometrial environment is also of interest in horses, where it is thought to regulate sperm–neutrophil interactions [143] and to modulate persistent mating-induced endometritis by suppressing the expression of proinflammatory cytokines in the endometrium [144].

## 5. Effects of Seminal Plasma on Endometrial Microenvironment in Pathological Conditions

### 5.1. Effects of Abnormal Seminal Plasma on Endometrial Microenvironment

#### 5.1.1. Advanced Male Age

A male age of over 40 years can have a significant impact on fertility and offspring health [145]. Functional decline in senile semen has recently been identified as another factor other than sperm that contributes to age-related declines in male fertility [146]. Wang et al. found that sExos changed with age and affected the uterine immune microenvironment in female mice, leading to reduced implantation rates [132]. The embryo implantation rate of female mice in the aged male mice SP-treated group was lower than that in the young male mice SP-treated group. RNA sequencing analyses showed that the levels of uterine DCs-associated cytokines and chemokines were altered in the aged male mice SP-treated group. The inhibitory effect on DC maturation was weaker in the aged SP than in the young SP (Table 2). Meanwhile, young sExos partially restored the decrease in implantation rate in the aged group, suggesting that age-related alterations in sExos may mediate the decrease in implantation rate in the aged SP group through uterine immunomodulation. These findings provide new ideas for clinical semen-assisted therapy [132].

#### 5.1.2. Male High-Fat Diet (HFD)

Non-genomic transmission of paternal effects on offspring is thought to result from genetic and epigenetic alterations in sperm DNA due to current and past environmental exposures and other events [10,147]. However, there is growing evidence that the effects of paternal exposures are also transmitted to offspring through alterations in SP [2,8,9,148,149,150]. Currently, the prevalence of obesity in men of reproductive age is increasing. It is widely recognized that male obesity is associated with low fertility, a prolonged time to conception, and the need for assisted reproductive technology to conceive [151]. To observe the altered composition of SP in obese men and its effects on the female immune response, Schjenken et al. evaluated the composition of seminal vesicular fluid and its immunomodulatory function in a patrilineal obese mouse model [152]. It was found that the concentration of TGF-β key isoforms (TGF-β1, TGF-β2, and TGF-β3) in the SP of mice fed a high-fat diet (HFD) was significantly reduced, as well as several other cytokines associated with the regulation of the immune response of females after mating, including CCL3, C-C motif chemokine ligand 11 (CCL11), CXCL1, IL-1β, IL-6, IL-17, and TNF. And altered semen composition in HFD male mice was associated with altered endometrial gene expression and attenuated Treg responses in females after mating. These results suggest that HFD and the subsequent metabolic state of obesity can alter the physiology and secretion of male accessory glands, thereby affecting female immune adaptations to pregnancy (Table 2). Schjenken et al. suggest that studies of the mechanisms of the paternal role in conception may be beneficial for the development of new therapeutic targets to protect fertility and reproductive outcomes [152].

**Table 2:** Effects of abnormal SP on endometrial microenvironment and reproductive outcome under pathological conditions.

Pathological Status	Species	Changes in SP	Outcomes	References
SP from advanced male age	Mouse	Age-related alterations in sExos	Weakened the inhibitory effect on DC maturation Decreased the embryo implantation rate in the uterus of mating female mice	[132]
SP from HFD male	Mouse	Reduced TGF-β, CCL3, CCL11, CXCL1, IL-1β, IL-6, IL-17, TNF	Altered endometrial gene expression and attenuated Treg responses in females after mating Affected mating female immune adaptations to pregnancy	[152]
SP from LPD male	Mouse	Unclear	Inhibited uterine inflammatory responses and affected vascular remodeling in mating females Affected offspring metabolic health	[149]

Abbreviations: CCL3, C-C motif chemokine ligand 3; CCL11, C-C motif chemokine ligand 11; CXCL1, C-X-C motif chemokine ligand 1; DC, dendritic cell; HFD, high-fat diet; IL, interleukin; LPD, low-protein diet; sExos, SP exosomes; SP, seminal plasma; TGF-β, transforming growth factor-β; TNF, tumor necrosis factor; Treg, regulatory T cell.

#### 5.1.3. Male Low-Protein Diet (LPD)

HFD-induced paternal obesity and diabetes result in impaired embryo development [153]. Similarly, paternal malnutrition significantly affects embryonic metabolism, fetal growth, and adult cardiometabolic health [154,155,156]. Mechanisms by which paternal diet affects offspring health may include alterations in testicular and sperm epigenetic regulation, SP composition, and the maternal reproductive tract responses that regulate early embryonic development [149]. Watkins et al. [149] found that females mated with low-protein diet (LPD) males showed significant reductions in uterine proinflammatory cytokines and chemokines, including reduced levels of TNF, IL-1β, GCSF, CCL3, and IFN-γ, as well as a decreased expression of genes related to PGs synthesis pathways, and significant decreases in the area and circumference of uterine blood vessels. Paternal LPD was associated with offspring obesity, metabolic dysfunction, and altered gut microbiota. The results suggest that paternal LPD inhibits normal maternal uterine inflammatory responses, affects vascular remodeling, and adversely affects embryonic development and offspring metabolic health (Table 2). This study links poor paternal diet to semen quality, pre-implantation uterine immunity, and offspring health. In addition, Watkins et al. have presented interesting insights into the SP microbiota [149]. SP has its own microbiota, but it remains to be determined whether this microbiota influences the maternal reproductive tract microbiota, thereby altering the gut microbiota of the offspring at birth. This may be another mechanism by which the father’s diet influences the health and metabolism of his offspring.

### 5.2. Pathological Changes of Abnormal Endometrium Exposed to SP

#### 5.2.1. Endometriosis

Endometriosis is a common gynecological condition characterized by the presence of functional endometrial tissue outside the uterine cavity, which can cause pelvic pain and affect fertility, affecting approximately 6–10% of women worldwide [157]. The exact pathogenesis of endometriosis has not been determined [158]. Sampson’s theory suggests that endometriotic implants originate from endometrial tissue traveling retrogradely through the fallopian tubes into the peritoneal cavity during menstruation [159]. The cyclic regeneration of endometrial tissue is associated with mesenchymal stem cells (MSCs) [160], and endometrial MSCs have been detected in the endometrial basal lamina and endometriotic implants [161]. SP can promote endometriotic growth and pathologic development [162] by inducing epithelial–mesenchymal transdifferentiation and the expression of myofibroblastic metaplasia markers in endometriotic cells [163] and activating the growth of MSCs in endometriotic implants [164]. TGF-β1 in SP plays a key role in this process and directly regulates the proliferation of MSCs by activating MAPK and phosphatidylinositol 3-kinase (PI3K)/protein kinase B (PKB, also known as AKT) pathways [164].

#### 5.2.2. Endometritis

Endometritis is an inflammatory disease involving the lining of the uterus, which may affect the zygote implantation and lead to infertility or miscarriage [165]. The causative factors of endometritis are complex and are mainly caused by bacteria such as Staphylococcus, Escherichia coli, and Streptococcus [166]. Transient endometritis that occurs after mating is normal and can be caused by infectious factors (bacteria and fungi) or non-infectious factors (sperm) [167]. In species such as horses, pigs, and dogs, the lack of a cervical barrier and the deposition of large amounts of semen in the uterus are more likely to interfere with the uterine immune response and microbiome, resulting in persistent endometritis [168]. SP exhibits both anti-inflammatory and pro-inflammatory properties in regulating endometritis. On the one hand, SP induces the secretion of inflammatory cytokines from endometrial tissues and enhances pro-inflammatory responses [144]. On the other hand, there are also CRISP-3 and lactoferrin in SP with anti-inflammatory effects, which can regulate the secretion of anti-inflammatory cytokines and resist copulation-induced endometritis [144,169]. In general, SP exerts mainly pro-inflammatory effects on endometrial tissues. The endometrium shows a rapid increase in pro-inflammatory cytokines and an upregulation of inflammation-regulating cytokines upon exposure to SP [170]. However, persistent endometritis leads to a failure to remove the inflammation in a timely manner and an imbalance in the expression of pro- and anti-inflammatory cytokines in the endometrium, which ultimately affects the success of pregnancy [170].

### 5.3. Other Pathological Conditions

One study suggested a possible link between human papillomavirus (HPV) infection in sperm and idiopathic recurrent pregnancy loss (RPL). The prevalence of HPV sperm infection was significantly higher in RPL-affected couples than in fertile couples, with approximately one in five patients having an HPV infection in their semen samples [171]. Other disorders, such as antiphospholipid syndrome (APS), are associated with alterations in endometrial angiogenesis, placental defects, and fetal loss [172]. The effect of SP on endometrial angiogenesis in APS deserves further investigation. In addition, the endometrium has its microbiota, which may affect endometrial receptivity. However, the activity of the endometrial microbiome and the possible effect of “reproductive tract dysbiosis” on fertility is still unclear [173]. Further research is needed to clarify whether SP microbiota and its changes affect endometrial microbiota biodiversity and the impact on subsequent fertility.

At present, although there are many studies on the effects of SP on the endometrial immune microenvironment under physiological conditions, the research on the influence of various abnormal SP on the endometrium is limited to one to two articles and there are few diseases involved. Future studies on the interaction between SP and endometrium in pathological conditions should be increased to find new factors leading to clinically adverse pregnancy outcomes and possible therapeutic targets. Notably, the interaction of sperm with neutrophils in utero may help to activate an adaptive immune response to antigens in semen [9]. However, it is still unclear whether abnormal sperm affects the microenvironment of endometrium and the interaction between SP and endometrium. The relevant studies need to be conducted.

## 6. Conclusions and Prospect

**Figure 2:**
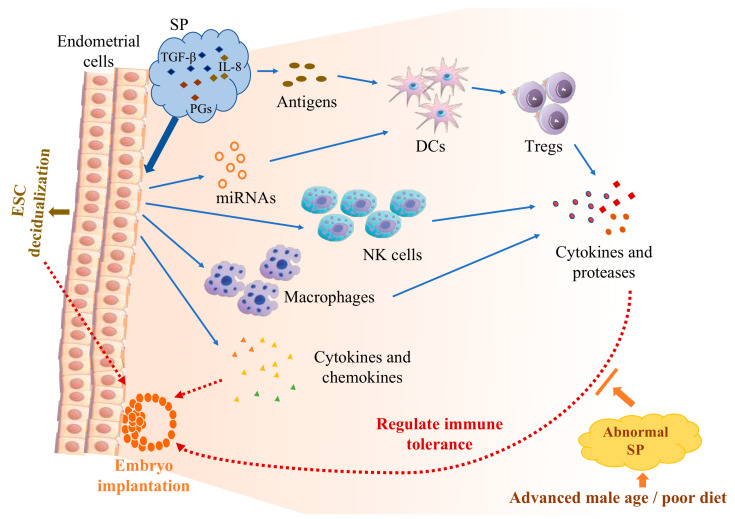
The endometrial microenvironment responds to the immune regulation of seminal plasma (SP). Key signaling factors in SP, including transforming growth factor (TGF)-β, prostaglandins (PGs), and interleukin (IL)-8, interact with endometrial epithelial cells and endometrial stromal cells (ESCs). They can induce the secretion of pro-inflammatory cytokines and chemokines and promote ESC decidualization and the changes in the number and phenotype of natural killer (NK) cells and macrophages. Antigens in the SP can be presented by dendritic cells (DCs), driving the activation and expansion of regulatory T cells (Tregs), which recognize and respond to paternal antigens. Changes in miRNAs in uterine tissue caused by SP also contribute to regulating DC and Treg function. Cytokines and proteases secreted by immune cells are conducive to the establishment of maternal immune tolerance to the embryo. Together, these changes promote embryo implantation. However, advanced age or poor diet in men can lead to abnormal changes in SP, which interfere with women’s immune adaptation to pregnancy and negatively affect embryo implantation.

Under physiological conditions, SP transports sperm into the female reproductive tract and also affects the endometrial microenvironment. It can cause changes in the phenotype of immune cells, induce the secretion of pro-inflammatory cytokines and chemokines, and promote endometrial decidualization, which are all conducive to conception and embryo implantation. Among the signaling factors that play a key role in SP are TGF-β, PGs, IL-8, and exosomes. However, abnormal SP can disrupt the normal immune response of a woman’s uterus, affecting embryo implantation and even offspring health (Figure 2). In turn, the pathologic state of the uterus can respond negatively to SP, further promoting pathological progression and affecting reproductive outcomes. Thus, signaling factors of SP are involved in regulating the endometrial immune microenvironment. The specific mechanisms of it need to be explored in depth.

Currently, there is increasing interest in male factors contributing to adverse pregnancy outcomes, but most studies have focused on the effects of spermatozoa. In the future, studies on the effects of abnormal SP on the reproductive tract immune microenvironment such as female endometrium and pregnancy outcomes will help to provide new ideas and intervention targets for the diagnosis and treatment of clinical infertility. In particular, the impact of SP on offspring health deserves more attention. Although the involvement of SP is usually not required for embryo culture and transfer during clinical assisted reproduction therapy, identifying the signaling factors of SP that are conducive to implantation and pregnancy in physiological states and intervening in the endometrium during assisted reproductive therapy may be an effective way to increase the success rate of assisted reproduction and improve pregnancy outcomes.

## Figures and Tables

**Table 1:** Immunoregulation of main components of SP on endometrial microenvironment under physiological conditions.

SP Components	Species	Endometrial Cells or Tissues	Monitoring Indicators	Outcomes	References
TGF-β	Mouse	Endometrial epithelial cells	Upregulated: GM-CSF	Induced proinflammatory cytokine and chemokine synthesis in the endometrium	[89,90,96]
	Human	Ect1 cervical epithelial cells	Upregulated: GM-CSF, IL-1	Induced proinflammatory cytokine synthesis in the endometrium	[118]
PGs	Human	Endometrial epithelial cells, DCs	Upregulated: FGF-2, COX-2, VEGF, EGFR, ERK 1/2 signaling pathways (endometrial epithelial cells); IL-10, TGF-β (DCs) Downregulated: IL-12p70, IL-1β, TNF-α, IL-6 (DCs)	Promoted endometrial inflammatory response Induced angiogenesis Promoted the differentiation of tolerogenic DCs	[125,126]
IL-8	Human	Endometrial epithelial cells	Upregulated: *IL-1β*, *IL-6*, *LIF*	Stimulated the expression of proinflammatory cytokines	[128]
SP + P_4_	Human	ESCs	Upregulated: PRL, IGFBP1	Promoted the decidualization of ESCs Enhanced endometrial receptivity	[47]
MVs	Human	eSFs	Upregulated: IL-11	Promoted the decidualization of eSFs in women with PCOS and endometriosis	[52]
SF-EVs	Human	ESCs	Upregulated: PRL	Enhanced ESC decidualization	[53]
SP (unclear specific component)	Bovine	Endometrial epithelial cells, ESCs	Upregulated: *GM-CSF, IL-8, TGFB1, PTGS2, AKR1C4* (endometrial epithelial cells); *GM-CSF, IL1B, IL6, IL-8, IL17A, TGFB1, PTGS2, AKR1C4* (ESCs)	Modulated the expression of inflammatory mediators in the endometrium Altered the maternal environment of early pregnancy	[100]
	Pig	Endometrial tissue, uterine horn	Upregulated: *GM-CSF, IL-6, MCP-1, COX-2* (endometrial tissue) Downregulated: *PTGS2* (uterine horn)	Programmed the trajectory of uterine cytokine expression and leukocyte trafficking during early pregnancy, modulated the immune–cytokine network of the female reproductive system Regulated pre-implantation embryo development	[101,112]
	Horse	Endometrial biopsy	Upregulated: *IL-1B, IL-6, TNF-α, COX-2*	Caused an inflammatory endometrial response	[102]
	Sheep	Endometrial epithelial cells	Upregulated: GM-CSF, IL-8	Induced uterine inflammatory response	[103]
	Mouse	γδT cells	Upregulated: IL-17A	Regulated uterine inflammation	[104]

Abbreviations: AKR1C4, aldo-keto reductase family 1 member C4; COX-2, cyclooxygenase-2; DCs, dendritic cells; EGFR, epidermal growth factor receptor; ERK, extracellular-signal-regulated kinase; ESCs, endometrial stromal cells; eSFs, endometrial stromal fibroblasts; FGF-2, fibroblast growth factor 2; GM-CSF, granulocyte-macrophage colony stimulating factor; IGFBP1, insulin-like growth factor binding protein 1; IL, interleukin; LIF, leukemia inhibitory factor; MCP-1, monocyte chemoattractant protein 1; MVs, microvesicles; P4, progesterone; PCOS, polycystic ovary syndrome; PGs, prostaglandins; PRL, prolactin; PTGS2, prostaglandin-endoperoxide synthase 2; SF-EVs, seminal fluid extracellular vesicles; SP, seminal plasma; TGF-β, transforming growth factor-β; TGFB1, transforming growth factor beta 1; TNF-α, tumor necrosis factor-α; VEGF, vascular endothelial growth factor.

## Data Availability

No new data were created or analyzed in this study. Data sharing is not applicable to this article.
